# Age-related differences in clinical characteristics and survival in lung cancer

**DOI:** 10.3389/fonc.2026.1868163

**Published:** 2026-08-10

**Authors:** Huan-yu Wang, Ting-ting Chen, Yu-jie Zhong, Wei Zhao, Hao Yao, Li-jie Ma

**Affiliations:** 1 Department of Pulmonary and Critical Care Medicine, The General Hospital of Western Theater Command, Chengdu, Sichuan, China; 2 Department of Respiratory and Critical Care Medicine, The Affiliated Hospital of Southwest Medical University, Luzhou, Sichuan, China; 3 Department of Basic Faculty, Engineering University of PAP, Xi’an, Shaanxi, China; 4 Chengdu Medical College Clinical Medical College, Chengdu, Sichuan, China; 5 Department of Reproductive Medicine, The 924th Hospital of Joint Logistics Support Force of PLA, Guilin, Guangxi, China; 6 Reproductive Medicine Center, The General Hospital of Western Theater Command, Chengdu, Sichuan, China; 7 Institute of Basic Medicine, North Sichuan Medical College, Nanchong, Sichuan, China; 8 Affiliated Hospital of Southwest Jiaotong University, The General Hospital of Western Theater Command, Chengdu, Sichuan, China; 9 Tissue Stress Injury and Functional Repair Key Laboratory of Sichuan Province, Chengdu, Sichuan, China

**Keywords:** age differences, lung cancer, nutritional–inflammatory indices, overall survival, prognostic factors, progression-free survival

## Abstract

**Background:**

Lung cancer predominantly affects older adults; however, an increasing number of cases are diagnosed in younger patients. Age-related differences in baseline characteristics, survival outcomes, and prognostic factors remain incompletely understood. This study aimed to compare clinical features, laboratory profiles, and survival outcomes between young and elderly patients with lung cancer and to identify prognostic factors associated with overall survival (OS) and progression-free survival (PFS).

**Methods:**

This retrospective cohort study screened patients with pathologically confirmed primary lung cancer diagnosed in 2022. Patients aged ≤45 years were classified as the young group, and those aged ≥75 years as the elderly group. Baseline demographic characteristics, laboratory parameters, and nutritional–inflammatory indices were compared between groups. Survival outcomes were evaluated using Kaplan–Meier analyses and compared with the log-rank test. Univariate and multivariate Cox proportional-hazards regression analyses were performed to identify prognostic factors for OS and PFS.

**Results:**

A total of 44 patients were included (21 young, 23 elderly). Elderly patients had higher smoking exposure and worse nutritional and inflammatory profiles, including lower albumin and prognostic nutritional index values. Younger patients showed significantly longer OS, while PFS was numerically longer but not statistically significant despite a tendency toward more advanced disease. In univariate analyses, histology type, gene mutation status, and treatment modality were associated with survival outcomes. In the simplified multivariable Cox models, immunotherapy was independently associated with improved OS, and targeted therapy, surgery, and immunotherapy were associated with better PFS.

**Conclusions:**

Young and elderly patients with lung cancer exhibit distinct clinical, laboratory, and survival profiles. Younger patients demonstrated more favorable genomic profiles and survival outcomes. These findings highlight the importance of age-specific evaluation and suggest that treatment-related and host-related factors should be considered in prognostic assessment.

## Highlights

- Younger lung cancer patients show better OS than elderly patients.
- Elderly patients have worse nutritional and inflammatory profiles.
- Smoking exposure is more common in elderly patients.
- Treatment modality, including targeted therapy, surgery, and immunotherapy, were associated with improved PFS.
- Immunotherapy is the sole independent protective factor for OS.

## Introduction

Lung cancer remains the leading cause of cancer-related mortality worldwide, accounting for a substantial proportion of global cancer deaths despite advances in early detection and treatment (1). The burden of lung cancer is disproportionately concentrated in older populations, reflecting cumulative exposure to carcinogens and age-related biological vulnerability (2). With population aging, the number of elderly patients diagnosed with lung cancer continues to increase, posing significant challenges in clinical decision-making, treatment tolerance, and prognostic assessment (3, 4). At the same time, lung cancer in younger individuals represents a distinct and increasingly recognized clinical entity. Although relatively rare, early-onset lung cancer often exhibits unique epidemiological, pathological, and molecular features, including a higher prevalence of adenocarcinoma and targetable genomic alterations, as well as a lower association with tobacco exposure (5–7). These contrasting age-related patterns underscore the heterogeneity of lung cancer across the lifespan and highlight the need for age-specific evaluation.

Previous studies have demonstrated that age is an important prognostic factor in lung cancer, although the direction and magnitude of its impact remain inconsistent across disease stages and treatment modalities (8, 9). Elderly patients are frequently underrepresented in clinical trials and are less likely to receive guideline-concordant therapy, contributing to worse observed outcomes (10, 11). In contrast, younger patients often receive more aggressive treatment but may present with more advanced disease at diagnosis, potentially offsetting the survival advantage conferred by better functional status (12, 13). Recent real-world and registry-based analyses have further highlighted age-related differences in treatment patterns, response to systemic therapy, and survival outcomes, particularly in the era of immunotherapy and targeted therapy (14–16). However, direct comparisons between young and elderly patients that comprehensively incorporate clinical characteristics, laboratory parameters, and nutritional–inflammatory indices remain limited. Age-related differences in treatment patterns have also been reported across disease stages and histological subtypes, with elderly patients less likely to receive chemotherapy or multimodal therapy and younger patients more frequently treated with intensive systemic approaches, contributing to disparities in real-world outcomes (10, 16, 17).

Nutritional status and systemic inflammation have emerged as important prognostic determinants in lung cancer, influencing treatment tolerance, immune response, and survival outcomes (18, 19). Indices such as albumin level, prognostic nutritional index, and inflammatory ratios have shown prognostic value across age groups, yet their interaction with age-related survival differences has not been fully elucidated. Given the growing recognition of lung cancer heterogeneity by age and the clinical implications of aging, a clearer understanding of age-specific characteristics and prognostic factors is needed. Therefore, the present study aimed to perform a retrospective comparative analysis of young and elderly patients with lung cancer, focusing on baseline clinical characteristics, laboratory and nutritional–inflammatory profiles, survival outcomes, and prognostic factors for overall and progression-free survival. By elucidating age-related differences, this study seeks to provide evidence to support more individualized risk stratification and management strategies across the age spectrum.

## Methods

### Patients

This retrospective cohort study included patients with pathologically confirmed primary lung cancer who were diagnosed and treated at a single tertiary medical center between January 1 and December 31, 2022. Patients were screened from the institutional lung cancer database and stratified by age into two predefined cohorts: a young group (≤45 years) and an elderly group (≥75 years). Patients aged 45–75 years were excluded to ensure clear age separation. Additional exclusion criteria included incomplete clinical records, presence of other concurrent malignancies, chronic inflammatory or autoimmune diseases, institutional administrative restrictions, and absence of available survival data. The final study population consisted of 44 patients, including 21 young and 23 elderly patients.

### Ethical statement

This study was conducted in accordance with the Declaration of Helsinki and was approved by the Institutional Review Board of the participating institution. Given the retrospective design and the use of anonymized clinical data, the requirement for written informed consent was waived.

### Treatment and clinical management

Patients received standard-of-care management according to tumor stage, histological subtype, molecular characteristics, and overall clinical condition, as determined by a multidisciplinary team. Treatment modalities included surgical resection, systemic therapy, or best supportive care, when appropriate. Treatment decisions were individualized, particularly for elderly patients, with consideration of performance status, comorbid conditions, and patient preference. Given the observed differences in treatment allocation across clinical presentations and age groups, individual treatment modalities (targeted therapy, surgery, immunotherapy, and radiotherapy) were included as separate covariates in the survival analyses to adjust for potential treatment-related confounding.

### Data collection

Baseline demographic and clinical variables were extracted from electronic medical records, including age, sex, smoking history, clinical symptoms, comorbid conditions, histological subtype, and clinical stage at diagnosis. Laboratory parameters obtained at baseline included carcinoembryonic antigen, total protein, albumin, red blood cell count, platelet count, and inflammatory markers. Nutritional–inflammatory indices, such as the prognostic nutritional index and lymphocyte-to-monocyte ratio, were calculated using standard formulas described in previous studies.

### Follow-up

Patients were followed from the date of diagnosis until death or the last follow-up. Follow-up assessments were conducted in accordance with institutional practice and included clinical evaluation, imaging studies, and laboratory tests. Overall survival was defined as the time from diagnosis to death from any cause or last follow-up. Progression-free survival was defined as the time from diagnosis to disease progression, recurrence, death, or last follow-up, whichever occurred first.

### Statistical analysis

Continuous variables were evaluated for distributional characteristics using visual inspection and Kolmogorov-Smirnov tests. They were summarized as means with standard deviations or medians with interquartile ranges, and compared using Student’s t-test or the Mann–Whitney U test, as appropriate. Categorical variables were presented as frequencies and percentages and compared using the chi-square test or Fisher’s exact test. Because patients with incomplete baseline records or who were lost to follow-up (N = 52) were excluded prior to the primary analysis, complete-case analyses were conducted without the need for data imputation.

Survival outcomes were estimated using the Kaplan–Meier method and compared with the log-rank test. Cox proportional-hazards regression models were used to identify prognostic factors for overall and progression-free survival. Importantly, the proportional hazards assumption for all Cox models was thoroughly evaluated using Schoenfeld residuals, and no systematic violations were observed. Given the limited number of events and sparse distribution of several molecular variables, multivariable Cox models were carefully simplified by excluding rare mutation subtypes and limiting covariates to clinically robust factors to prevent model overfitting. Individual treatment modalities (targeted therapy, surgery, immunotherapy, and radiotherapy) were entered separately into the simplified Cox regression models as adjustment covariates to account for treatment-related confounding and were not interpreted as causal determinants of survival outcomes. Finally, given that this observational study is explicitly exploratory and hypothesis-generating, formal adjustments for multiple comparisons were not applied to avoid inflating Type II errors and masking potentially meaningful clinical signals. A two-sided P value < 0.05 was considered statistically significant; however, all reported P values are unadjusted and must be interpreted with caution.

## Results

### Baseline demographic and clinical characteristics

Baseline demographic and clinical characteristics were compared between the young and elderly lung cancer cohorts (**Table 1**). By design, age differed significantly between groups (P < 0.001). Sex distribution was similar between cohorts.

**Table 1:** Baseline characteristics of young and elderly patients with lung cancer.

Variable	Young(n=21)	Elderly(n=23)	P
Age (years)	40.143 ± 3.759	79.391 ± 3.928	<0.001***
Gender			0.242
Male	11 (52.4%)	16 (69.6%)	
Female	10 (47.6%)	7 (30.4%)	
Histological Type			0.359
Adenocarcinoma	18 (85.7%)	18 (78.3%)	
Squamous cell	1 (4.8%)	4 (17.4%)	
Small cell lung cancer	2 (9.5%)	1 (4.3%)	
Clinical Stage			0.339
I	0	3 (13.0%)	
II	2 (9.5%)	1 (4.3%)	
III	4 (19.0%)	3 (13.0%)	
IV	14 (66.7%)	16 (69.6%)	
Smoking History			0.036**
No	12 (57.1%)	6 (26.1%)	
Yes	9 (42.9%)	17 (73.9%)	
Gene Mutation Status			0.031**
Wild type or missing	3 (14.3%)	12 (52.2%)	
EGFR	9 (42.9%)	8 (34.8%)	
ALK	7 (33.3%)	1 (4.3%)	
KRAS	1 (4.8%)	0	
ROS1	1 (4.8%)	0	
ERBB2	0	1 (4.3%)	
RET	0	1 (4.3%)	
Treatment Modality			0.015**
Chemotherapy	2 (9.5%)	7 (30.4%)	
Targeted	14 (66.7%)	7 (30.4%)	
Surgery	4 (19.0%)	1 (4.3%)	
Immunotherapy	1 (4.8%)	7 (30.4%)	
Radiotherapy	0	1 (4.3%)	
Dyspnea			0.065*
No	18 (85.7%)	14 (60.9%)	
Yes	3 (14.3%)	9 (39.1%)	

Clinical stage was defined according to the eighth edition of the TNM staging system. *P < 0.10; **P < 0.05; ***P < 0.01.

Adenocarcinoma was the predominant histological subtype in both groups, with a higher proportion observed among younger patients, whereas squamous cell carcinoma and small cell lung cancer were more common in elderly patients; however, no significant difference in histological distribution was observed. Stage IV disease was most common in both groups (66.7% vs 69.6%, P = 0.339).

The elderly group had a higher prevalence of smoking history (73.9% vs 42.9%, P = 0.036). Treatment distribution differed significantly (P = 0.015), with targeted therapy predominating in younger patients (66.7%), while chemotherapy and immunotherapy were more common in the elderly (both 30.4%). Gene mutation profiles also differed (P = 0.031), with higher frequencies of ALK (33.3% vs 4.3%) and EGFR mutations (42.9% vs 34.8%) in younger patients, whereas wild-type status predominated in the elderly (52.2% vs 14.3%). Dyspnea was more frequent in the elderly (39.1% vs 14.3%), showing a borderline significance (P = 0.065) (**Table 1**).

### Baseline laboratory parameters and nutritional–inflammatory indices

Baseline laboratory parameters and nutritional–inflammatory indices differed between the young and elderly lung cancer cohorts (**Table 2**). Levels of carcinoembryonic antigen were significantly higher in elderly patients than in younger patients (P < 0.001). Elderly patients also exhibited lower total protein and albumin levels (P = 0.003 and P = 0.004, respectively), along with reduced red blood cell and platelet counts (P = 0.047 and P = 0.018).

**Table 2:** Baseline laboratory parameters and nutritional–inflammatory ratios in young and elderly patients with lung cancer.

Variable	Young(n=21)	Elderly(n=23)	P
Carcinoembryonic Antigen	10.500 ± 5.916	31.500 ± 6.494	<0.001***
CA199	10.500 ± 5.916	31.000 ± 6.205	<0.001***
CA125	194.105 ± 352.419	53.024 ± 76.036	0.087*
Total Protein	71.890 ± 5.741	67.067 ± 3.580	0.003***
Albumin	44.519 ± 4.023	40.771 ± 3.973	0.004***
Red Blood Cell Count	4.564 ± 0.653	4.206 ± 0.484	0.047**
Platelet Count	254.905 ± 80.707	201.455 ± 60.566	0.018**
Monocyte Percentage	6.833 ± 1.752	8.341 ± 3.216	0.065*
HALP	34.19 ± 15.85	34.62 ± 14.72	0.925
NPAR	1.59 ± 0.19	1.74 ± 0.34	0.087
PNI	51.38 ± 4.41	46.88 ± 4.09	0.001***
NLR	4.37 ± 3.11	5.63 ± 5.04	0.331
PLR	193.36 ± 57.42	187.75 ± 101.00	0.824
LMR	3.00 ± 1.15	2.33 ± 1.01	0.048**
SII	1124.72 ± 743.37	1137.40 ± 1187.92	0.967
PIV	680.68 ± 757.69	749.67 ± 1.72.11	0.808

Data are presented as mean ± standard deviation.

HALP denotes hemoglobin–albumin–lymphocyte–platelet score; NPAR, neutrophil percentage–to–albumin ratio; PNI, prognostic nutritional index; NLR, neutrophil–to–lymphocyte ratio; PLR, platelet–to–lymphocyte ratio; LMR, lymphocyte–to–monocyte ratio; SII, systemic immune-inflammation index;

PIV, pan-immune-inflammation value.*P < 0.10; **P < 0.05; ***P < 0.01.

In contrast, prognostic nutritional index values were significantly higher in younger patients (P = 0.003), while lymphocyte-to-monocyte ratio was lower in elderly patients (P = 0.048). No significant differences were observed between groups with respect to CA125, HALP score, neutrophil-to-lymphocyte ratio, platelet-to-lymphocyte ratio, or other inflammatory markers (**Supplementary Table 2**).

### Patient selection and study cohort

**Figure 1:**
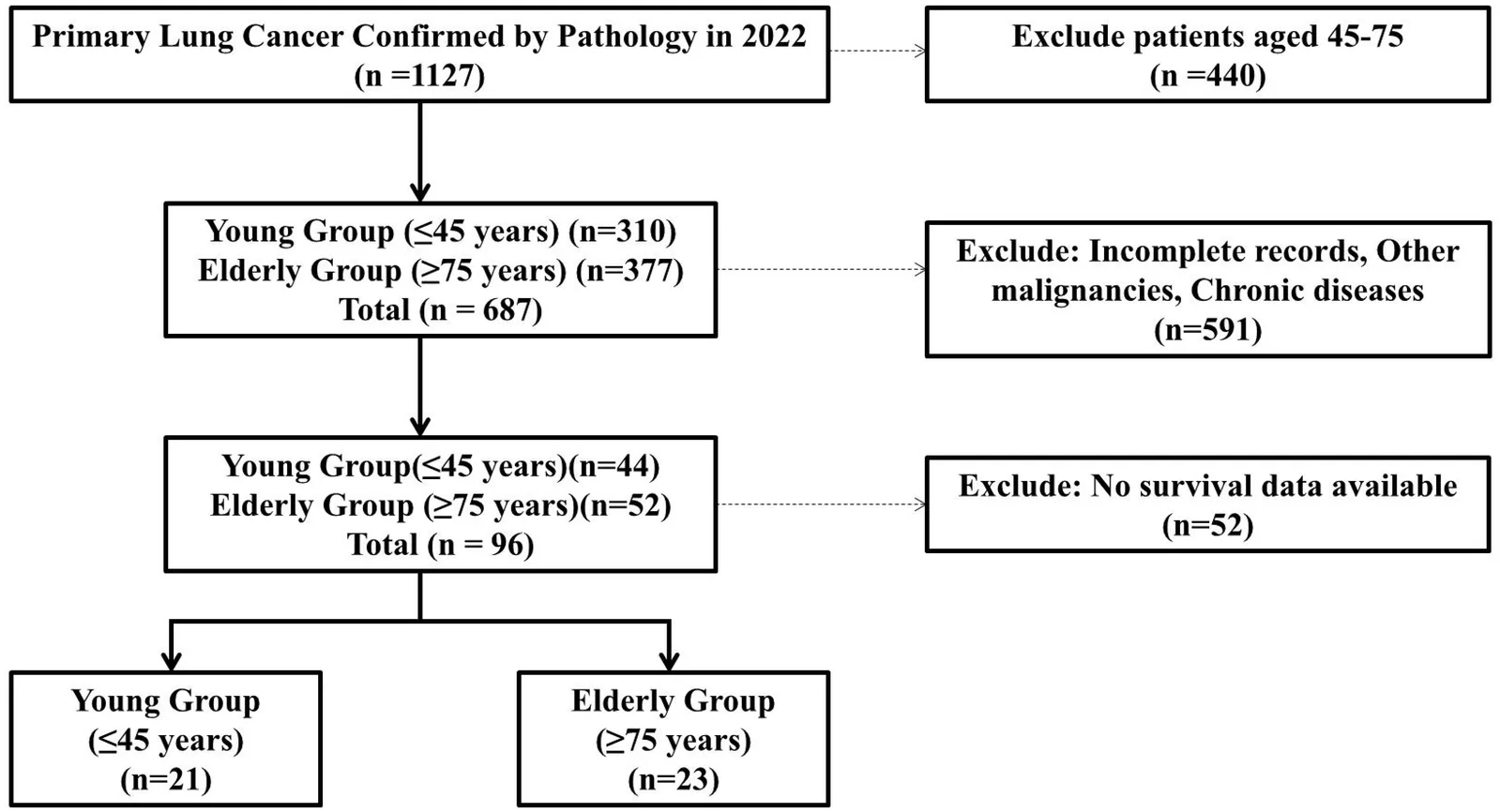
Flow diagram of patient selection and study cohort formation. A total of 1,127 patients with pathologically confirmed primary lung cancer diagnosed in 2022 were initially assessed for eligibility. Patients aged 45–75 years were excluded. After additional exclusions for incomplete records, other malignancies, chronic diseases, institutional administrative restrictions, and unavailable survival data, 44 patients were included in the final analysis, comprising 21 young patients (≤45 years) and 23 elderly patients (≥75 years).

A total of 1,127 patients with pathologically confirmed primary lung cancer diagnosed in 2022 were initially screened (**Figure 1**). Patients aged 45–75 years were excluded, leaving 687 patients who met the predefined age criteria and were categorized into a young group (≤45 years) and an elderly group (≥75 years). After further exclusions due to incomplete medical records, coexisting malignancies, chronic diseases, or institutional administrative restrictions, 96 patients remained eligible for analysis.

Patients without available survival data were subsequently excluded. The final study cohort consisted of 44 patients, including 21 young patients and 23 elderly patients, who were included in the final analysis (**Figure 1**).

### Survival outcomes according to age group

**Figure 2:**
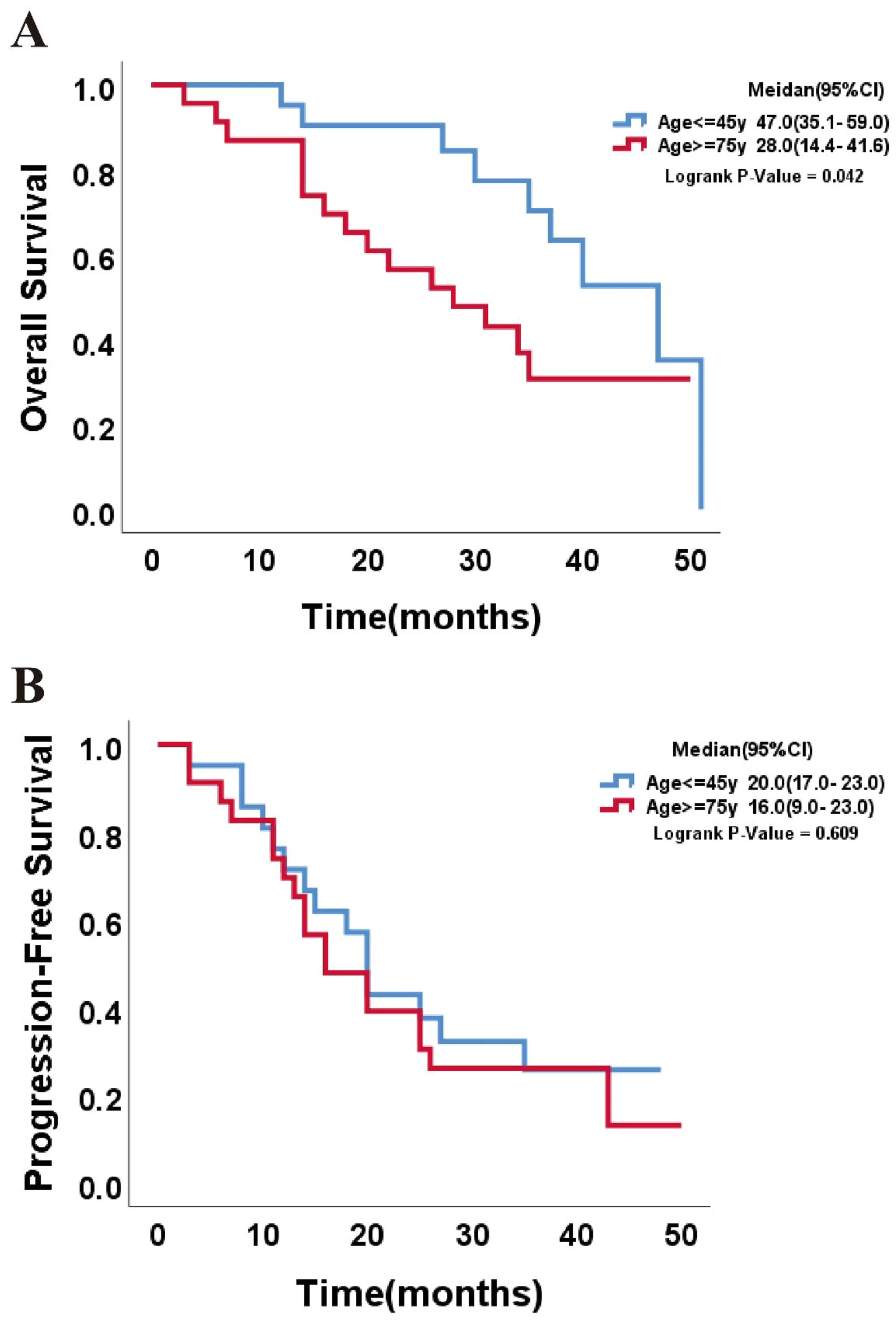
Kaplan–Meier analysis of overall survival and progression-free survival according to age group. Panel **(A)** shows overall survival among young patients (≤45 years) and elderly patients (≥75 years). Panel **(B)** shows progression-free survival in the two age-defined cohorts. Survival distributions were estimated using the Kaplan–Meier method, and differences between groups were compared with the log-rank test.

Kaplan–Meier analysis showed that median overall survival (OS) was significantly longer in patients aged ≤45 years than in those aged ≥75 years (47.0 vs 28.0 months; log-rank P = 0.042). Median progression-free survival (PFS) was also longer in younger patients (20.0 vs 16.0 months), but the difference was not statistically significant (P>0.05) (**Figure 2**). Given the small sample size (n=44) and limited number of PFS events, this nonsignificant result may reflect limited statistical power.

**Figure 3:**
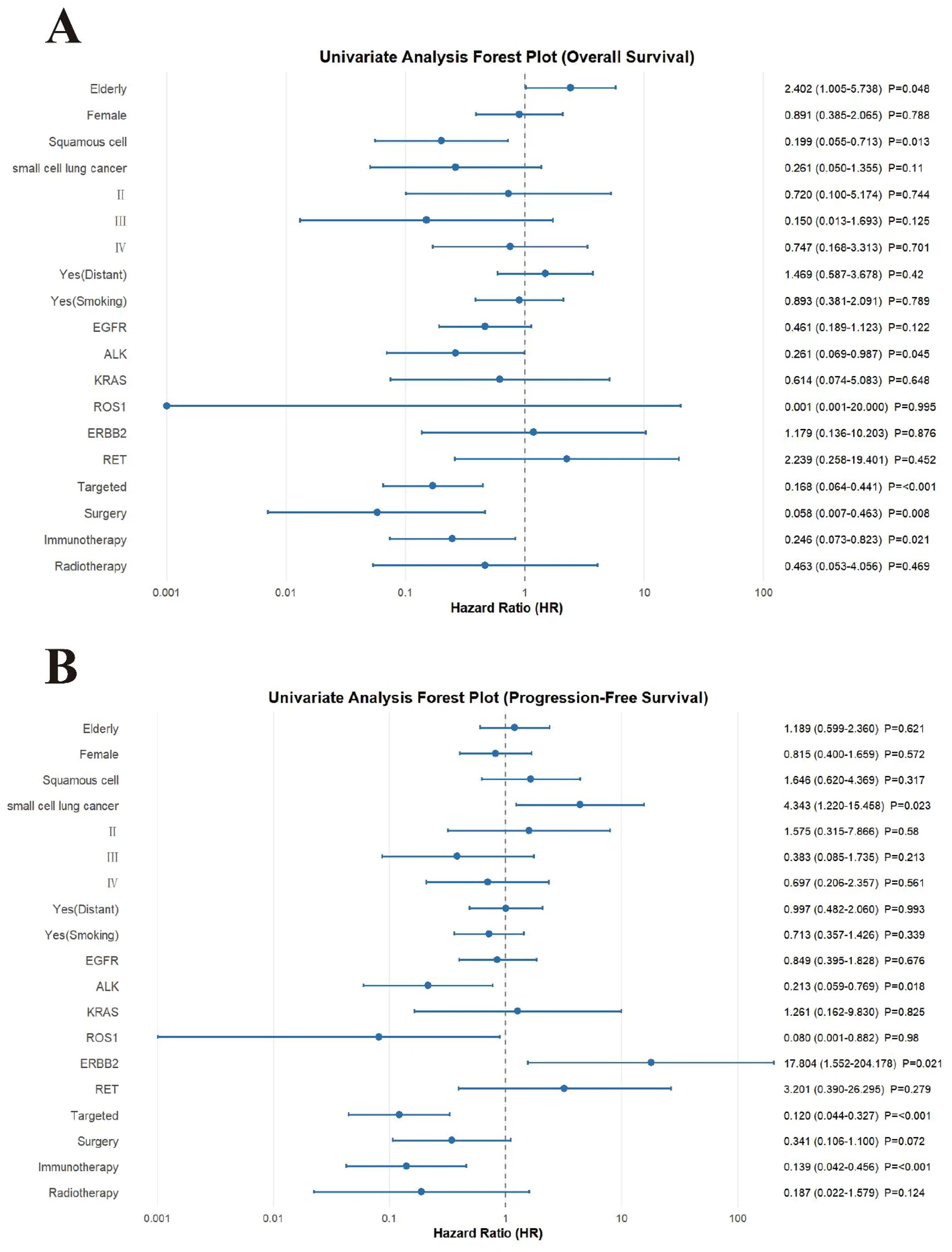
Forest plots of univariate Cox regression analyses for overall survival and progression-free survival. Panel **(A)** shows hazard ratios and 95% confidence intervals for factors associated with overall survival. Panel **(B)** shows hazard ratios and 95% confidence intervals for factors associated with progression-free survival. Hazard ratios were estimated using univariate Cox proportional-hazards models.

**Figure 4:**
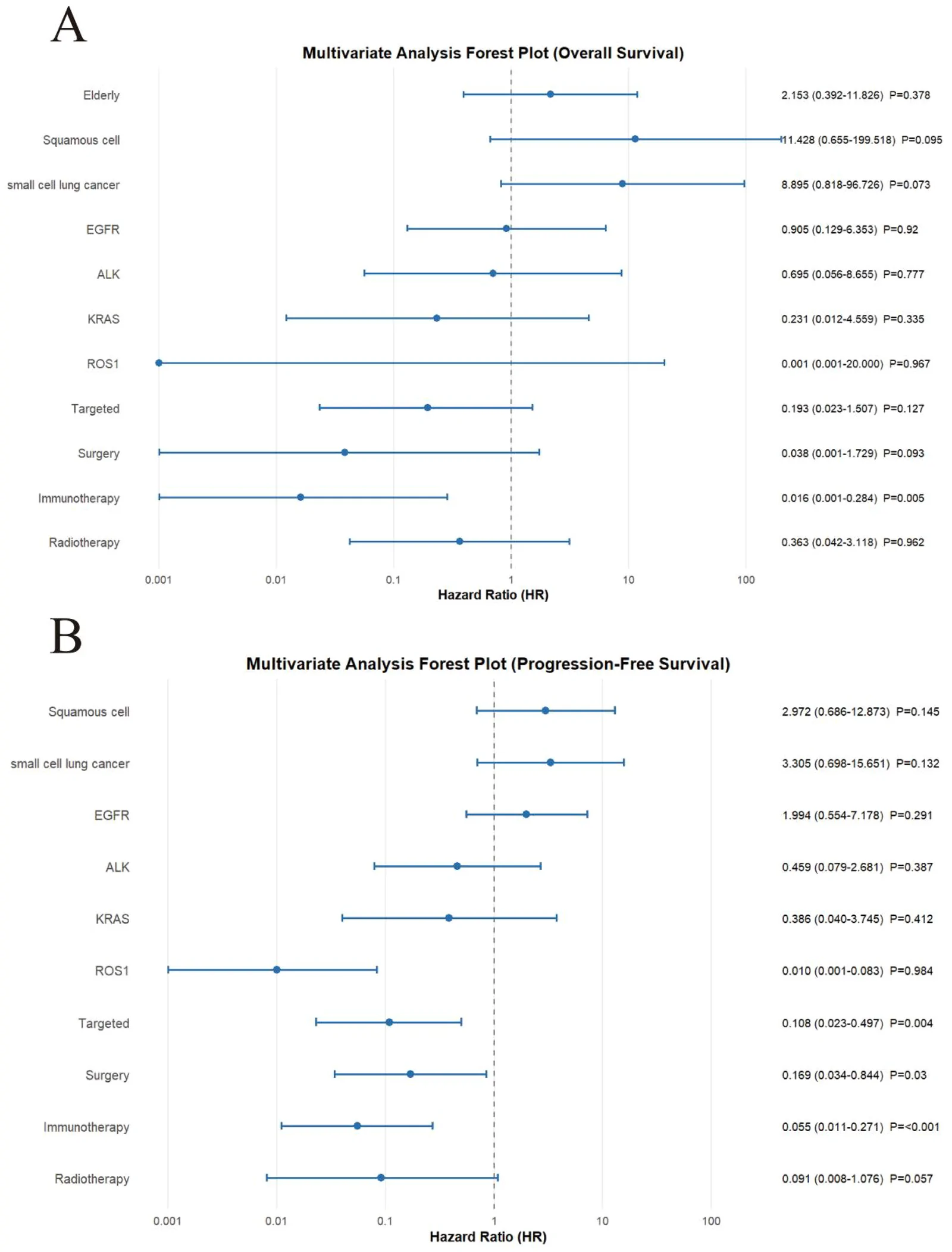
Forest plots of multivariate Cox regression analyses for overall survival and progression-free survival. Panel **(A)** shows adjusted hazard ratios and 95% confidence intervals for factors independently associated with overall survival. Panel **(B)** shows adjusted hazard ratios and 95% confidence intervals for factors independently associated with progression-free survival. Hazard ratios were estimated using multivariate Cox proportional-hazards models adjusting for clinically relevant covariates.

Treatment was significantly associated with overall survival (OS), with immunotherapy (HR = 0.246, 95% CI: 0.073–0.823, P = 0.021), targeted therapy (HR = 0.168, 95% CI: 0.064–0.441, P<0.001), and surgery (HR = 0.058, 95% CI: 0.007–0.463, P = 0.008) associated with reduced mortality. Age (HR = 2.402, P = 0.048), histology (HR = 0.199, P = 0.013), and ALK mutation (HR = 0.261, P = 0.045) were also associated with OS, whereas sex, clinical stage, distant metastasis, smoking history, and other mutations were not (**Figure 3A**). Immunotherapy was independently associated with improved OS in multivariable analysis (HR = 0.016, 95% CI: 0.001–0.284, P = 0.005) (**Figure 4A**). Other variables and selected nutritional–inflammatory indices are detailed in **Supplementary Table 3** (**Table 3**).

**Table 3:** Simplified multivariate Cox regression analyses for overall survival (OS).

Variable	Category/comparison	Univariate HR (95%CI)	P value	Multivariate HR (95%CI)	P value
Age Group	≥75 vs ≤45 years	2.402 (1.005-5.738)	0.048**	2.153 (0.392-11.826)	0.378
Histology	Squamous cell vs Adenocarcinoma	0.199 (0.055-0.713)	0.013**	11.428 (0.655-199.518)	0.095*
Gene Mutation	ALK vs Wild type	0.261 (0.069-0.987)	0.045**	0.695 (0.056-8.655)	0.777
Targeted therapy	Yes vs No	0.168 (0.064-0.441)	<0.001***	0.193 (0.023-1.507)	0.127
Surgery	Yes vs No	0.058 (0.007-0.463)	0.008***	0.038 (0.001-1.729)	0.093*
Immunotherapy	Yes vs No	0.246 (0.073-0.823)	0.021	0.016 (0.001-0.284)	0.005

Hazard ratios (HRs) and 95% confidence intervals (CIs) were estimated using Cox proportional-hazards models.

Given the limited number of events, multivariable models were restricted to clinically essential variables to avoid overfitting.

PNI was included only in univariate analysis.

*P < 0.10; **P < 0.05; ***P < 0.01.

### Prognostic factors for progression-free survival

Univariate and multivariate Cox proportional-hazards regression analyses were performed to evaluate factors associated with progression-free survival (**Table 4**). In univariate analyses, histological type, selected gene mutation variables, and treatment modality were associated with PFS; however, rare mutation subtypes showed unstable estimates because of sparse distribution. Therefore, rare mutation subtypes, including RET and ERBB2, were excluded from the final multivariable model. , 

**Table 4:** Simplified univariate and multivariable Cox regression analyses for progression-free survival (PFS).

Variable	Category/comparison	Univariate HR(95%CI)	P value	Multivariate HR(95%CI)	P value
Age Group	≥75 vs ≤45 years	1.189( 0.599-2.360)	0.621	—	—
Histology	SCLC vs Adenocarcinoma	4.343 (1.220-15.458)	0.023**	3.305 (0.698-15.651)	0.132
Gene Mutation	ALK vs Wild type	0.213 (0.059-0.769)	0.018**	0.459 (0.079-2.681)	0.387
Surgery	Yes vs No	0.341 (0.106-1.100)	0.072	0.169 (0.034-0.844)	0.03**
Targeted	Yes vs No	0.120 (0.044-0.327)	<0.001***	0.108 (0.023-0.497)	0.004***
Immunotherapy	Yes vs No	0.139 (0.042-0.456)	<0.001***	0.055 (0.011-0.271)	<0.001***

Hazard ratios (HRs) and 95% confidence intervals (CIs) were estimated using Cox proportional-hazards models.

Multivariable models were restricted to clinically essential covariates due to limited event numbers to minimize overfitting.

Rare mutation subtypes, including RET and ERBB2, were excluded from the final multivariable model because of sparse-data instability.

Treatment modalities were modeled as separate covariates in the multivariable Cox regression models.

*P < 0.10; **P < 0.05; ***P < 0.01.

Histology was associated with progression-free survival (PFS), with small cell lung cancer showing a higher risk of progression or death than adenocarcinoma (HR = 4.343, 95% CI: 1.220–15.458, P = 0.023). ALK (HR = 0.213, P = 0.018) and ERBB2 mutations (HR = 17.804, P = 0.021) were also associated with PFS. Targeted therapy (HR = 0.120, P<0.001) and immunotherapy (HR = 0.139, P<0.001) were associated with reduced risk, whereas age, sex, stage, distant metastasis, smoking, and other mutations were not (**Figure 3B**).

Multivariable analysis showed that targeted therapy (HR = 0.108, 95% CI: 0.023–0.497, P = 0.004), surgery (HR = 0.169, 95% CI: 0.034–0.844, P = 0.030), and immunotherapy (HR = 0.055, 95% CI: 0.011–0.271, P<0.001) were independently associated with improved PFS (**Figure 4B**). No other prognostic factors showed independent associations (**Supplementary Table 4**).

## Discussion

In this retrospective cohort study, we observed age-related differences in clinical characteristics and treatment patterns among patients with lung cancer. Younger patients had a higher prevalence of targetable mutations (particularly ALK and EGFR) and were more likely to receive targeted therapy, whereas elderly patients exhibited higher smoking exposure, poorer nutritional and inflammatory profiles, and a greater proportion of wild-type tumors. Survival analysis demonstrated that younger patients had significantly longer overall survival than elderly patients (P<0.05), and age was associated with survival in univariate analysis. However, this association was not retained after multivariable adjustment, possibly reflecting the limited sample size and confounding by differences in treatment allocation, with younger patients more frequently receiving targeted therapy and elderly patients more often treated with immunotherapy and chemotherapy. In our simplified multivariable analyses, treatment modality was significantly associated with survival outcomes (P<0.05); however, this primarily reflects the adjustment for baseline clinical heterogeneities rather than causal treatment effects. Furthermore, the unexpected and clinically counterintuitive hazard ratios observed for clinical stage lacked statistical significance. This pattern is primarily attributable to sparse-data bias driven by the highly unbalanced stage distribution—specifically, a predominance of advanced-stage cases and a severely limited number of early-stage patients. Consequently, these stage-specific estimates reflect statistical instability rather than true clinical effects and must be interpreted with caution. Overall, these findings highlight substantial heterogeneity across age groups, suggesting that differences in baseline health status, genomic profiles, and treatment patterns may contribute to divergent survival outcomes.

The results of the present study are largely consistent with previous reports describing age-related heterogeneity in lung cancer characteristics and outcomes. Prior epidemiological and registry-based studies have shown that younger patients more frequently present with adenocarcinoma and experience better survival than elderly patients, despite differences in disease stage and treatment patterns (6, 12, 13). Population-based analyses have similarly identified increasing age as an adverse prognostic factor in lung cancer (8, 9). In contrast, studies focusing on elderly populations have emphasized the influence of comorbidities, undertreatment, and reduced treatment adherence on survival outcomes, which may partly explain poorer prognosis in older patients (10, 11, 16). Although age has been reported as an independent prognostic factor for overall survival in several large-scale analyses (8, 14), age did not retain independent significance in our multivariable models, potentially reflecting differences in cohort composition and the inclusion of nutritional–inflammatory parameters. Emerging evidence linking nutritional status and systemic inflammation to lung cancer prognosis, particularly among elderly patients, supports the importance of host-related factors in interpreting age-associated survival differences (18, 19).

Several methodological aspects distinguish this study from prior age-stratified analyses. By applying strict age cutoffs and excluding intermediate-age patients, we enhanced biological and clinical contrast between young and elderly populations, an approach variably adopted in earlier studies (6, 12, 13). In addition to conventional clinicopathological variables, we incorporated baseline laboratory measures and nutritional–inflammatory indices, which have increasingly been recognized as prognostic markers but are infrequently integrated into age-comparative survival analyses (8, 18). Younger patients have been reported to harbor a higher prevalence of targetable genomic alterations and to exhibit differential responses to targeted therapies and immunotherapy, including epidermal growth factor receptor–tyrosine kinase inhibitors and immune checkpoint inhibitors, which may contribute to improved survival in contemporary cohorts (5, 14, 15). Prior studies have also suggested that age influences treatment responsiveness and toxicity in these therapeutic contexts, potentially modifying survival trajectories across age groups (16). By integrating clinical characteristics, laboratory-derived indices, and survival analyses within a single cohort, our study provides a more comprehensive assessment of age-related heterogeneity than registry-based or treatment-focused analyses alone.

Several limitations of this study should be acknowledged. First, the retrospective design may have introduced selection bias and limits causal inference, a limitation common to observational studies evaluating age-related survival differences in lung cancer (8, 9). The exclusion of patients aged 45–75 years to enhance age contrast may introduce a degree of selection bias and may limit the generalizability of the findings, as the study focuses on more distinct age groups. Second, the relatively small sample size and single-center setting may restrict generalizability and reduce statistical power, particularly in multivariable analyses. Furthermore, our multivariable analyses were constrained by the relatively small sample size and limited number of survival events. To prevent severe model overfitting, we could not comprehensively adjust for several clinically important prognostic confounders—specifically performance status, comorbidity burden, exact smoking intensity (e.g., pack-years), metastatic burden, and the completeness of molecular testing. Although individual treatment covariates were included to partially account for differences in treatment allocation and baseline clinical decision-making, residual confounding remains inevitable. Consequently, attributing survival differences purely to age or treatment in this cohort remains challenging, and our findings must be interpreted strictly as exploratory and hypothesis-generating. Additionally, 52 patients were excluded due to loss to follow-up and unavailability of survival outcomes, which may introduce potential selection bias. Third, importantly, treatment assignment in this retrospective cohort was strongly influenced by confounding by indication. Treatment modalities differed significantly across age groups, reflecting real-world clinical decisions based on baseline characteristics, performance status, and mutation profiles rather than randomized protocols (4, 10, 11). Consequently, the hazard ratios associated with treatment variables in our simplified multivariable models are intended solely to adjust for baseline confounding and must not be interpreted as causal estimates of treatment efficacy. In addition, molecular profiling data were not systematically available, precluding assessment of age-related differences in genomic alterations known to influence prognosis and therapeutic response, especially in younger patients (5, 6, 13). Although baseline nutritional and inflammatory indices were evaluated, longitudinal changes in these parameters were not assessed, despite growing evidence supporting their prognostic relevance over time (23). Finally, similar age-related disparities have been reported across diverse populations and study designs, including very young adults, pediatric and adolescent cohorts, and elderly-specific assessments, underscoring the need for age-sensitive analytical frameworks in lung cancer research (18, 20–22).

## Conclusions

Age-related heterogeneity in lung cancer extends beyond chronological classification and encompasses differences in clinical presentation, host condition, and survival trajectories. This study reinforces the concept that younger and elderly patients represent distinct clinical populations, shaped by variations in baseline functional status, nutritional and inflammatory profiles, and disease burden. Survival outcomes appear to be influenced by the combined effects of histological type, treatment modality and host-related factors, underscoring the limitations of age-based stratification alone. Incorporating simple, routinely available indicators of nutritional status and systemic inflammation into clinical assessment may enhance prognostic evaluation and support more individualized management across age groups. For elderly patients in particular, comprehensive evaluation of host vulnerability may be critical for optimizing therapeutic decision-making and supportive care, while younger patients warrant continued attention to biological diversity and long-term disease implications. Further research integrating clinical, molecular, and host-related parameters, along with real-world treatment patterns, may refine risk stratification and inform age-sensitive strategies aimed at improving outcomes for patients with lung cancer.

## Data Availability

The raw data supporting the conclusions of this article will be made available by the authors, without undue reservation.

## Supplementary material

Supplementary Table 1 Additional baseline characteristics.

Supplementary Table 2 Additional baseline laboratory parameters.

Supplementary Table 3 Additional univariate cox regression analyses for overall survival.

Supplementary Table 4 Additional univariate cox regression analyses for progression-free survival.
