# Supplementary material for: Age-related differences in clinical characteristics and survival in lung cancer
Source: Front Oncol. 2026 Aug 10;16:1868163. doi: 10.3389/fonc.2026.1868163 (PMC13500315; doi:10.3389/fonc.2026.1868163)
Supplement: Supplementary file 1 [file Table1.docx]

| **Supplementary Table 1. Additional Baseline Characteristics** | | | |
| --- | --- | --- | --- |
|  | Young（n=21） | Elderly（n=23） | P |
| Distant Metastasis |  |  | 0.837 |
| No | 7（33.3%） | 7（30.4%） |  |
| Yes | 14（66.7%） | 16（69.6%） |  |
| Cough |  |  | 0.355 |
| No | 5（23.8%） | 3（13.0%） |  |
| Yes | 16（76.2%） | 20（87.0%） |  |
| Sputum Production |  |  | 0.139 |
| No | 11（52.4%） | 7（30.4%） |  |
| Yes | 10（47.6%） | 16（69.6%） |  |
| Weight Loss |  |  | 0.202 |
| No | 18（85.7%） | 16（69.6%） |  |
| Yes | 3（14.3%） | 7（30.4%） |  |
| Dyspnea |  |  | 0.223 |
| No | 14（66.7%） | 19（82.6%） |  |
| Yes | 7（33.3%） | 4（17.4%） |  |
| Chest Pain |  |  | 0.334 |
| No | 21（100.0%） | 22（95.7%） |  |
| Yes | 0 | 1（4.3%） |  |
| Hemoptysis |  |  | 0.587 |
| No | 17（81.0%） | 20（87.0%） |  |
| Yes | 4（19.0%） | 3（13.0%） |  |

Footnote: Data are presented as n (%).

P values were calculated using the chi-square test or Fisher’s exact test, as appropriate.

| **Supplementary Table 2. Additional Baseline Laboratory Parameters** | | | |
| --- | --- | --- | --- |
|  | Young（n=21） | Elderly（n=23） | P |
| Alpha-fetoprotein | 2.879±1.675 | 2.927±1.245 | 0.918 |
| Fibrinogen | 3.913±1.214 | 4.269±1.180 | 0.336 |
| Creatinine | 67.714±17.822 | 76.048±20.711 | 0.17 |
| White Blood Cell Count | 7.681±3.426 | 7.931±4.669 | 0.843 |
| Hemoglobin | 131.095±22.396 | 131.591±23.095 | 0.943 |
| Hematocrit | 40.229±5.886 | 39.391±3.463 | 0.576 |
| Neutrophil Percentage | 70.652±8.506 | 70.123±10.909 | 0.86 |
| Lymphocyte Percentage | 19.638±6.037 | 18.073±7.737 | 0.465 |
| Eosinophil Percentage | 2.324±2.066 | 2.845±3.135 | 0.525 |
| Basophil Percentage | 0.552±0.346 | 0.618±0.359 | 0.544 |
| Neutrophil Count | 5.589±3.190 | 5.866±4.392 | 0.815 |
| Lymphocyte Count | 1.372±0.405 | 1.225±0.432 | 0.256 |
| Monocyte Count | 0.520±0.247 | 0.604±0.294 | 0.322 |
| Eosinophil Count | 0.135±0.120 | 0.192±0.192 | 0.253 |
| Basophil Count | 0.041±0.027 | 0.043±0.023 | 0.818 |

Footnote: Data are presented as mean ± standard deviation.

P values were calculated using Student’s t test or Mann–Whitney U test, as appropriate.

| **Supplementary Table 3. Additional Univariate Cox Regression Analyses for Overall Survival** | | |
| --- | --- | --- |
|  | Univariate HR(95%CI) | P |
| Cough |  |  |
| No | 1 | NA |
| Yes | 1.130(0.408-3.123) | 0.825 |
| Sputum Production |  |  |
| No | 1 | NA |
| Yes | 1.548(0.578-4.149) | 0.319 |
| Weight Loss |  |  |
| No | 1 | NA |
| Yes | 1.494(0.566-3.969) | 0.433 |
| Dyspnea |  |  |
| No | 1 | NA |
| Yes | 1.699(0.664-4.399) | 0.216 |
| Chest Pain |  |  |
| No | 1 | NA |
| Yes | 0.922(0.348-2.440) | 0.874 |
| Fever |  |  |
| No | 1 | NA |
| Yes | 0.044(0.000-253.573) | 0.479 |
| Hemoptysis |  |  |
| No | 1 | NA |
| Yes | 0.385(0.115-1.286) | 0.2 |
| HALP | 0.996(0.970-1.022) | 0.761 |
| NPAR | 0.484(0.102-2.294) | 0.361 |
| PNI | 0.999(0.916-1.089) | 0.983 |
| NLR | 0.938(0.837-1.052) | 0.274 |
| PLR | 0.998(0.993-1.003) | 0.417 |
| LMR | 0.855(0.594-1.232) | 0.4 |
| SII | 1.000(0.999-1.000) | 0.316 |
| PIV | 1.000(0.999-1.000) | 0.912 |

Footnote: HR denotes hazard ratio; CI, confidence interval.

“No” was used as the reference category for categorical variables.

| **Supplementary Table 4. Additional Univariate Cox Regression Analyses for Progression-Free Survival** | | |
| --- | --- | --- |
|  | Univariate HR(95%CI) | P |
| Cough |  |  |
| No | 1 | NA |
| Yes | 0.744(0.322-1.719) | 0.489 |
| Sputum Production |  |  |
| No | 1 | NA |
| Yes | 0.939(0.470-1.876) | 0.858 |
| Weight Loss |  |  |
| No | 1 | NA |
| Yes | 1.716(0.794-3.708) | 0.169 |
| Dyspnea |  |  |
| No | 1 | NA |
| Yes | 1.798(0.859-3.764) | 0.12 |
| Chest Pain |  |  |
| No | 1 | NA |
| Yes | 0.912(0.409-2.034) | 0.822 |
| Fever |  |  |
| No | 1 | NA |
| Yes | 0.044(0.100-6.495) | 0.401 |
| Hemoptysis | 1 | NA |
| No | 0.451(0.158-1.288) | 0.137 |
| Yes | 0.996(0.974-1.018) | 0.729 |
| HALP | 0.996(0.974-1.018) | 0.729 |
| NPAR | 0.553(0.156-1.962) | 0.359 |
| PNI | 1.023 (0.950-1.101) | 0.547 |
| NLR | 0.985(0.909-1.066) | 0.702 |
| PLR | 1.000(0.996-1.004) | 0.986 |
| LMR | 0.786(0.570-1.083) | 0.141 |
| SII | 1.010(0.879-1.026) | 0.875 |
| PIV | 1.000(0.989-1.010) | 0.162 |

Footnote: HR denotes hazard ratio; CI, confidence interval.

“No” was used as the reference category for categorical variables.
